# Tobacco Use in Top-Grossing Movies — United States, 2010–2018

**DOI:** 10.15585/mmwr.mm6843a4

**Published:** 2019-11-01

**Authors:** Michael A. Tynan, Jonathan R. Polansky, Danielle Driscoll, Claire Garcia, Stanton A. Glantz

**Affiliations:** ^1^Office on Smoking and Health, National Center for Chronic Disease Prevention and Health Promotion, CDC; ^2^Onbeyond LLC, Fairfax, California; ^3^Breathe California Sacramento Region, Sacramento, California; ^4^University of California, San Francisco.

The Surgeon General has concluded that there is a causal relationship between depictions of smoking in movies and initiation of smoking among young persons (*1*). Youths heavily exposed to onscreen smoking imagery are more likely to begin smoking than are those with minimal exposure (*1*,*2*). To assess tobacco-use imagery in top-grossing youth-rated movies (General Audiences [G], Parental Guidance [PG], and Parents Strongly Cautioned [PG-13]),* 2010–2018 data from the Breathe California Sacramento Region and University of California-San Francisco’s Onscreen Tobacco Database were analyzed.^†^ The percentage of all top-grossing movies with tobacco incidents remained stable from 2010 (45%) to 2018 (46%), including youth-rated movies (31% both years). However, total tobacco incidents increased 57% from 2010 to 2018, with a 120% increase in PG-13 movies. Tobacco incidents in PG-13 fictional movies declined 57% from 511 in 2010 to an all-time low of 221 in 2018. Although the number of PG-13 fictional movies with tobacco incidents declined 40% during 2010–2018, the number of PG-13 biographical dramas with tobacco incidents increased 233%. In 2018, biographical dramas accounted for most tobacco incidents, including 82% of incidents in PG-13 movies; 73% of characters who used tobacco in these biographical dramas were fictional. Continued efforts could help reduce tobacco incidents in top-grossing movies, particularly in PG-13 biographical dramas, to help prevent youth smoking initiation.

Breathe California counts tobacco incidents, defined as the use or implied use of a tobacco product (i.e., cigarettes, cigars, pipes, hookahs, smokeless tobacco products, or electronic cigarettes), in U.S. top-grossing movies (movies ranking among the top 10 in theatrical gross receipts for at least 1 week), which account for 98% of U.S. movie ticket sales (*3*). Consistent with previous reports on this topic (*3*–*5*), this analysis is based upon assessments of movies for tobacco use by at least two independent monitors; any differences were resolved by a supervisor who independently assessed the movie using the same protocol.^§^

To calculate the percentage of movies with tobacco incidents, the number of movies with any tobacco incidents was divided by the total number of movies, and the average number of tobacco incidents per movie was calculated for each motion picture company. For each year during 2010–2018, the number of top-grossing movies with tobacco incidents and overall number of tobacco incidents were calculated. Results were also analyzed by Motion Picture Association of America ratings (G, PG, PG-13, and Restricted [R]). To identify movie type, production details in movie industry databases and trade publications were used to classify the top-grossing movies into three main genres: fiction, biographical dramas, and documentaries. The identity of each character using tobacco in biographical dramas was also examined to determine whether the character was fictional or an actual person.

In 2018, among the 139 top-grossing movies, 64 (46%) included tobacco incidents, compared with 62 (45%) of 137 in 2010. Among the 55 top-grossing R-rated movies, 38 (69%) had tobacco incidents in 2018, compared with 35 (71%) of 49 in 2010 (Table 1). Among youth-rated movies (G, PG, or PG-13), 26 (31%) of 84 had tobacco incidents in 2018, compared with 27 (31%) of 88 in 2010. During 2010–2018, the number of top-grossing movies with tobacco incidents was highest in 2013 (76) and lowest in 2014 (58).

**TABLE 1:** Number and percentage of top-grossing movies with tobacco incidents, number of tobacco incidents, and total number of top-grossing movies, by Motion Picture Association of America (MPAA) rating* and movie company — United States, 2010–2018

Movie company	MPAA rating	2010	2011	2012		2013	2014	2015	2016	2017	2018	Total
**Movies with tobacco incidents, no. (%)**												
Comcast (Universal)	G or PG	0 (0)	0 (0)	0 (0)		0 (0)	0 (0)	0 (0)	0 (0)	0 (0)	0 (0)	**0 (0)**
	PG-13	1 (17)	4 (40)	3 (50)		2 (29)	6 (67)	3 (30)	2 (18)	5 (56)	5 (38)	**31 (38)**
	R	6 (86)	6 (86)	8 (73)		10 (77)	5 (71)	5 (50)	2 (22)	6 (75)	3 (38)	**51 (64)**
Disney	G or PG	1 (11)	0 (0)	0 (0)		0 (0)	0 (0)	0 (0)	0 (0)	0 (0)	0 (0)	**1 (2)**
	PG-13	0 (0)	3 (60)	1 (33)		2 (40)	0 (0)	2 (50)	1 (20)	0 (0)	0 (0)	**9 (25)**
	R	0 (0)	1 (100)	0 (0)		1 (100)	0 (0)	0 (0)	0 (0)	0 (0)	0 (0)	**2 (100)**
Fox	G or PG	0 (0)	2 (29)	1 (17)		0 (0)	0 (0)	0 (0)	0 (0)	0 (0)	0 (0)	**3 (7)**
	PG-13	3 (38)	3 (50)	2 (40)		2 (33)	4 (57)	4 (36)	4 (67)	2 (40)	6 (75)	**30 (48)**
	R	5 (71)	2 (100)	3 (100)		6 (100)	5 (63)	5 (100)	4 (80)	6 (100)	7 (100)	**43 (88)**
Independents^†^	G or PG	3 (60)	0 (0)	1 (50)		2 (67)	1 (20)	2 (67)	1 (17)	1 (20)	2 (33)	**13 (34)**
	PG-13	6 (55)	6 (46)	12 (52)		10 (50)	9 (47)	10 (59)	6 (38)	13 (54)	6 (40)	**78 (49)**
	R	15 (83)	6 (67)	15 (68)		19 (83)	7 (58)	16 (70)	16 (70)	18 (82)	14 (61)	**126 (72)**
Sony	G or PG	0 (0)	1 (17)	1 (33)		1 (33)	2 (50)	1 (20)	0 (0)	0 (0)	1 (25)	**7 (22)**
	PG-13	8 (67)	7 (58)	6 (60)		4 (57)	5 (71)	3 (50)	3 (33)	3 (50)	3 (50)	**42 (56)**
	R	2 (67)	7 (78)	6 (75)		5 (83)	5 (83)	4 (100)	5 (100)	3 (50)	7 (100)	**44 (81)**
Time Warner (Warner Bros.)	G or PG	0 (0)	0 (0)	0 (0)		1 (100)	0 (0)	0 (0)	0 (0)	0 (0)	0 (0)	**1 (6)**
	PG-13	2 (22)	4 (33)	4 (44)		3 (27)	2 (25)	4 (50)	2 (20)	3 (43)	3 (30)	**27 (32)**
	R	4 (50)	3 (50)	5 (83)		3 (50)	3 (33)	6 (60)	4 (67)	5 (63)	4 (67)	**37 (57)**
Viacom (Paramount)	G or PG	0 (0)	3 (60)	0 (0)		0 (0)	0 (0)	0 (0)	0 (0	0 (0)	0 (0)	**3 (20)**
	PG-13	3 (75)	3 (50)	2 (40)		1 (25)	2 (25)	2 (67)	5 (56)	4 (80)	0 (0)	**22 (45)**
	R	3 (50)	1 (33)	3 (75)		4 (100)	2 (67)	2 (67)	4 (100)	3 (75)	3 (75)	**25 (71)**
**Subtotals of movies with tobacco incidents, by ratings**												
**All companies**	All G or PG	4 (11)	6 (14)	3 (11)	4 (21)		3 (12)	3 (13)	1 (4)	1 (5)	3 (13)	28 (12)
	All PG-13	23 (43)	30 (47)	30 (49)		24 (40)	28 (46)	28 (47)	23 (35)	30 (60)	23 (38)	239 (44)
	All youth-rated^§^	27 (31)	36 (37)	33 (37)		28 (35)	31 (36)	31 (38)	24 (26)	31 (38)	26 (31)	267 (34)
	R	35 (71)	26 (70)	40 (74)		48 (81)	27 (60)	38 (69)	35 (67)	41 (76)	38 (69)	328 (71)
**Subtotals for all companies**	**All ratings**	**62 (45)**	**62 (46)**	**73 (51)**		**76 (55)**	**58 (44)**	**69 (50)**	**59 (41)**	**72 (53)**	**64 (46)**	**595 (48)**
**No. of tobacco incidents**												
Comcast (Universal)	G or PG	0	0	0		0	0	0	0	0	0	**0**
	PG-13	19	78	39		53	173	11	266	407	573	**1,619**
	R	35	154	251		398	76	113	50	326	135	**1,538**
Disney	G or PG	10	0	0		0	0	0	0	0	0	**10**
	PG-13	0	148	102		57	0	123	6	0	0	**436**
	R	0	20	0		4	0	0	0	0	0	**24**
Fox	G or PG	0	3	2		0	0	0	0	0	0	**5**
	PG-13	96	174	205		3	101	150	145	90	327	**1,291**
	R	274	36	47		278	210	59	47	150	415	**1,516**
Independents^†^	G or PG	20	0	19		2	15	5	4	10	9	**84**
	PG-13	132	22	282		315	625	187	124	256	234	**2,177**
	R	582	216	720		511	559	456	887	1,316	572	**5,819**
Sony	G or PG	0	9	2		1	12	83	0	0	8	**115**
	PG-13	198	166	178		26	184	15	144	28	78	**1,017**
	R	33	537	246		155	225	156	579	172	360	**2,463**
Time Warner (Warner Bros.)	G or PG	0	0	0		5	0	0	0	0	0	**5**
	PG-13	4	106	265		309	16	30	40	26	29	**825**
	R	80	62	267		233	343	322	539	123	42	**2,011**
Viacom (Paramount)	G or PG	0	95	0		0	0	0	0	0	0	**95**
	PG-13	115	50	92		12	66	3	86	98	0	**522**
	R	226	4	166		217	34	30	246	139	86	**1,148**
**Subtotals of no. of tobacco incidents, by ratings**												
**All companies**	All G or PG	30	107	23		8	27	88	4	10	17	314
	All PG-13	564	744	1,163		775	1,165	519	811	905	1,241	7,901
	All youth-rated^§^	594	851	1,186		783	1,192	607	815	915	1,258	8,201
	R	1,230	1,029	1,697		1,796	1,447	1,136	2,348	2,226	1,610	14,519
**Subtotals for all companies**	**All ratings**	**1,824**	**1,880**	**2,883**		**2,579**	**2,639**	**1,743**	**3,163**	**3,141**	**2,868**	**22,720**
**Total no. of top grossing movies**												
**All companies**	**All ratings**	**137**	**134**	**143**		**138**	**132**	**137**	**143**	**136**	**139**	**1,239**

* MPAA, the trade organization that represents the six major movies studios, assigns ratings: G = General Audiences (all ages admitted); PG = Parental Guidance Suggested (some material may not be suitable for children); PG-13 = Parents Strongly Cautioned (some material may be inappropriate for children under 13); R = Restricted (under age 17 requires accompanying parent or adult guardian).

^†^ Independent movie companies include producer-distributors that are not members of MPAA, but regularly adhere to MPAA ratings and advertising rules.

^§^ Youth-rated movies include G, PG, and PG-13.

The total number of tobacco incidents in top-grossing movies increased by 57%, from 1,824 in 2010 to 2,868 in 2018. The number of tobacco incidents reached a low of 1,743 in 2015 before increasing to a high of 3,163 in 2016. The total number of tobacco incidents in G- or PG-rated movies decreased from 30 in 2010 to 17 in 2018. In contrast, tobacco incidents increased from 564 to 1,241 (120%) in PG-13 movies and from 1,230 to 1,610 (31%) in R-rated movies, compared with those in 2010.

From 2010 to 2018, changes in the number of tobacco incidents in youth-rated movies varied by movie company. During this period, tobacco incidents dropped from 10 to zero in movies from Disney and from 115 to zero in Viacom movies and declined from 198 to 86 in Sony movies. Tobacco incidents increased approximately 2,900% in Comcast movies (from 19 to 573), 600% in Time Warner movies (from four to 29), 200% in Fox movies (from 96 to 327), and 60% in movies from independent companies (from 152 to 243).

Among the 1,239 top-grossing movies during 2010–2018, 1,110 (90%) were fictional, 114 (9%) were biographical dramas, and 15 (1%) were documentaries. During the same period, 83% of all movies with tobacco incidents were fictional, 16% were biographical dramas, and 1% were documentaries. The number of fictional PG-13 movies with tobacco incidents declined 40%, from 20 in 2010 to a low of 12 in 2018 (Figure). However, PG-13 biographical dramas with tobacco incidents increased 233% during this period, from three in 2010 (13% of PG-13 movies) to 10 in 2018 (43%). In 2018, among 1,241 tobacco incidents in PG-13 movies, biographical dramas accounted for 1,019 (82%).

**FIGURE:**
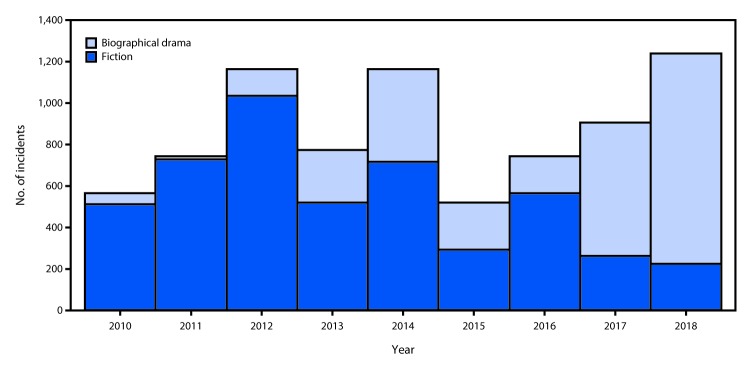
Number of tobacco incidents in PG-13–rated* movies, by genre^†^ — United States, 2010–2018 **Abbreviation:** PG-13 = Parents Strongly Cautioned (some material may be inappropriate for children under age 13 years). * Ratings are assigned by the Motion Picture Association of America, the trade organization that represents the six major movie studios. ^†^ Production details in movie industry databases and trade publications were used to classify the top-grossing movies as works of fiction or biographical dramas.

During 2010–2018, across rating categories, most tobacco users in biographical dramas were fictional characters, including 60% (three of five) in G- or PG-rated movies, 70% (213 of 306) in PG-13–rated movies, and 78% (355 of 455) in R-rated movies (Table 2). Biographical dramas accounted for 31% (766 of 2,505) of all characters shown using tobacco; however, 75% (571 of 766) of tobacco users in biographical dramas were fictional characters. In 2018, 73% (82 of 112) of characters who used tobacco in biographical dramas were fictional.

**TABLE 2:** Tobacco incidents and characters who use tobacco in top grossing biographical dramas, by Motion Picture Association of America (MPAA) rating* and movie company — United States, 2010–2018

Movie company	No. of biographical dramas	No. of movies with tobacco incidents (%)	No. of tobacco incidents	No. of characters who used tobacco	No. of fictional characters who used tobacco (%)
**G or PG rating**					
Comcast	1	0 (0)	0	0	0 (0)
Disney	3	0 (0)	0	0	0 (0)
Fox	2	0 (0)	0	0	0 (0)
Sony	6	2 (33)	93	3	1 (33)
Time Warner	1	0 (0)	0	0	0 (0)
Viacom	0	0 (0)	0	0	0 (0)
MPAA subtotal	13	2 (15)	93	3	1 (33)
Independents	3	1 (33)	10	2	2 (100)
**G or PG subtotal**	**16**	**3 (19)**	**103**	**5**	**3 (60)**
**PG-13 rating**					
Comcast	11	10 (91)	1,037	65	47 (72)
Disney	3	3 (100)	225	22	13 (59)
Fox	4	4 (100)	443	31	23 (74)
Sony	8	6 (75)	205	34	27 (79)
Time Warner	4	3 (75)	150	9	6 (67)
Viacom	2	2 (100)	71	13	11 (85)
MPAA subtotal	32	28 (88)	2,131	174	127 (73)
Independents	18	18 (100)	838	132	86 (65)
**PG-13 subtotal**	**50**	**46 (92)**	**2,969**	**306**	**213 (70)**
**Youth-rated (G, PG, and PG-13 combined)**					
Comcast	12	10 (83)	1,037	65	47 (72)
Disney	6	3 (50)	225	22	13 (59)
Fox	6	4 (67)	443	31	23 (74)
Sony	14	8 (57)	298	37	28 (76)
Time Warner	5	3 (60)	150	9	6 (67)
Viacom	2	2 (100)	71	13	11 (85)
MPAA subtotal	45	30 (67)	2,224	177	128 (72)
Independents	21	19 (90)	848	134	88 (65)
**Youth-rated subtotal**	**66**	**49 (74)**	**3,072**	**311**	**216 (69)**
**R rating**					
Comcast	9	7 (78)	464	56	48 (86)
Disney	1	1 (100)	4	1	0 (0)
Fox	5	5 (100)	202	25	19 (76)
Sony	1	1 (100)	147	35	30 (86)
Time Warner	9	9 (100)	849	89	63 (71)
Viacom	5	5 (100)	338	46	39 (85)
MPAA subtotal	30	28 (93)	2,004	252	199 (79)
Independents	18	16 (89)	1,276	203	156 (77)
**R subtotal**	**48**	**44 (92)**	**3,280**	**455**	**355 (78)**
**Total**	**114**	**93 (82)**	**6,352**	**766**	**571 (75)**

* Ratings are assigned by MPAA, the trade organization that represents the six major movies studios: G = General Audiences (all ages admitted); PG = Parental Guidance Suggested (some material may not be suitable for children); PG-13 = Parents Strongly Cautioned (some material may be inappropriate for children under 13); R = Restricted (under age 17 requires accompanying parent or adult guardian).

The use of electronic cigarettes, or vaping, appeared in 19 top-grossing movies during 2010–2018 (i.e., 2% of all movies and 3% of movies with tobacco incidents). Among these 19 movies, 15 were R-rated, and four were PG-13–rated.

## Discussion

Although the number of movies with tobacco incidents remained stable during 2010–2018, the number of tobacco incidents within these movies increased, including a 120% increase in PG-13 movies. Although the number of PG-13 fictional movies with tobacco incidents declined substantially during 2010–2018, the number of PG-13 biographical dramas with tobacco incidents approximately tripled. The total number of PG-13 movies in both these genres with tobacco incidents approximately doubled since 2010; approximately 80% of all tobacco incidents in 2018 occurred in PG-13 biographical dramas. These findings suggest that the increasing number of youth-rated biographical dramas with tobacco incidents has negated previous progress made in reducing tobacco incidents in youth-rated fictional movies.

All major motion picture companies have policies to reduce tobacco depictions in youth-rated movies^¶^; however, Disney and Viacom were the only companies with no tobacco use in youth-rated movies in 2018. Paid placement of tobacco brands is prohibited in media such as movies, television, and video games by the 1998 Master Settlement Agreement between states and tobacco companies.** Public health groups have suggested interventions to reduce tobacco imagery in movies, such as the Motion Picture Association of America assigning an R rating to any movie with tobacco imagery, unless it portrays an actual historical figure who used tobacco or depicts the negative effects of tobacco use (*6*–*8*). Research suggests that such an R rating, in coordination with additional interventions, could help eliminate tobacco incidents in youth-rated movies (*6*–*8*) and reduce youth cigarette smoking by an estimated 18% (*6*,*9*).

Establishing the impact of youths’ exposure to tobacco imagery through movies (as well as original programming on television, streaming and on-demand services, and social media) and the effects of this exposure on youths’ tobacco use is important. A recent survey of streaming content popular with young persons and analysis of two full seasons of 14 programs identified at least one tobacco incident in 86% of programs (*10*), even as tobacco incidents have begun to decline in fictional theatrical feature films. Reducing the reach of tobacco incidents in streaming and other media platforms is essential to protect youths from exposures that can normalize tobacco use. Continued research will be necessary to understand how this exposure affects youth tobacco initiation and use (*10*).

The findings in this report are subject to at least two limitations. First, detailed audience composition data are not publicly available, so the number of tobacco-use impressions delivered by a particular movie to specific populations (e.g., children and adolescents) could not be determined. Second, the measure to assess tobacco exposure from movies should be interpreted cautiously because movies can be viewed through other media platforms that do not contribute to the calculation of in-theater impressions (e.g., physical discs, broadcast or cable television, and video-on-demand services).

Tobacco related incidents in youth-rated movies remained common, particularly in biographical dramas. The majority of persons using tobacco in these biographical dramas were fictional, not historical, figures. Studios could limit tobacco use in biographical dramas to real persons who actually used tobacco. Other evidence-based solutions could be implemented by producers and distributors of youth-rated entertainment to reduce the public health risk caused by exposure to on-screen tobacco imagery. For example, assigning all movies with tobacco incidents an R rating could eliminate tobacco product imagery from youth-rated films, which could further reduce initiation of tobacco product use among U.S. youths.

**Summary**

**What is already known about this topic?**

The Surgeon General has concluded that there is a causal relationship between depictions of smoking in movies and the initiation of smoking among young persons.

**What is added by this report?**

From 2010 to 2018, tobacco incidents in top-grossing movies increased 57%, including a 120% increase in those rated PG-13. In 2018, biographical dramas accounted for most tobacco incidents, including 82% of those in PG-13 movies; 73% of characters who used tobacco in these biographical dramas were fictional.

**What are the implications for public health practice?**

Continued efforts are needed to reduce tobacco incidents in movies, particularly in PG-13–rated biographical dramas. Giving movies with tobacco incidents an R rating would eliminate tobacco product imagery from youth-rated films.

## Footnotes

* Ratings assigned by the Motion Picture Association of America (a trade organization that represents the major movie studios) include General Audiences (G): all ages admitted; Parental Guidance Suggested (PG): some material may not be suitable for children; Parents Strongly Cautioned (PG-13): some material may be inappropriate for children under 13; and Restricted (R): under 17 requires accompanying parent or adult guardian.

† https://smokefreemovies.ucsf.edu/.

§ Two common methods used to count tobacco use incidents in movies are to count the number of scenes in which tobacco use occurs or to count the number of cuts in which tobacco use occurs. In the second method, a new incident is counted each time 1) a tobacco product went off screen and then came back on screen; 2) a different actor was shown with a tobacco product; or 3) a scene changed and the new scene contained the use or implied use of a tobacco product. Incidents of implied use are rare and occur when a person is handed or is holding, but not necessarily, using a tobacco product. Despite the difference in methods, both yield consistent results and are valid for comparing the results across ratings, years, companies, etc.

¶ https://smokefreemovies.ucsf.edu/whos-accountable/company-policies.

** https://www.naag.org/assets/redesign/files/msa-tobacco/MSA.pdf.
